# Chamber‐based system for measuring whole‐plant transpiration dynamics

**DOI:** 10.1002/pei3.10094

**Published:** 2022-11-02

**Authors:** Alejandro Pieters, Marcus Giese, Marc Schmierer, Kristian Johnson, Folkard Asch

**Affiliations:** ^1^ Institute for Agricultural Sciences in the Tropics (Hans‐Ruthenberg Institute) University of Hohenheim Stuttgart Germany

**Keywords:** environmental drivers, gravimetry, transpiration, VPD, whole plant

## Abstract

Most of our insights on whole‐plant transpiration *(E)* are based on leaf‐chamber measurements using water vapor porometers, IRGAs, or flux measurements. Gravimetric methods are integrative, accurate, and a clear differentiation between evaporation and *E* can be made. Water vapor pressure deficit (VPD) is the driving force for *E* but assessing its impact has been evasive, due to confounding effects of other climate drivers. We developed a chamber‐based gravimetric method, in which whole plant response of *E* to VPD could be assessed, while keeping other environmental parameters at predetermined values. Stable VPD values (0.5–3.7 kPa) were attained within 5 min after changing flow settings and maintained for at least 45 min. Species differing in life form and photosynthetic metabolism were used. Typical runs covering the range of VPDs lasted up to 4 h, preventing acclimation responses or soilborne water deficit. Species‐specific responses of *E* to VPD could be identified, as well as differences in leaf conductance. The combined gravimetric‐chamber‐based system presented overcomes several limitations of previous gravimetric set ups in terms of replicability, time, and elucidation of the impact of specific environmental drivers on *E*, filling a methodological gap and widening our phenotyping capabilities.

## Graphical abstract

Most insights on transpiration (E) are based on leaf‐chamber measurements using water vapor porometers, IRGAs, or flux measurements. We developed a chamber‐based gravimetric method, to unequivocally assess plant response of E to VPD. The result is an affordable and reliable system that can be used to study the interaction between atmosphere and E at the whole‐plant level.

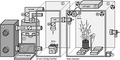

## 1 INTRODUCTION

Accurate measurement of leaf transpiration (*E*) and related stomatal conductance (*g*_*s*_) is fundamental to understanding plant energy dynamics and water relations at all scales, from pot to field (Pearcy et al., 2000). It is inextricably linked to photosynthesis as *E* and carbon assimilation share the same route in their way out of and into the leaf (Stanhill, 1986). It is a major component of the hydrological cycle and is a useful reflection of the adjustments of a plant to the surrounding environment. Available methods to measure *E* in herbaceous plants include: (1) porometers or infrared gas analyzers (IRGAs) that measure either at the single leaf or whole‐plant level; (2) lysimeters that measure intact soil column weight loss (usually combining *E* and evaporation of individual plants or smaller communities) (Bello & Van Rensburg, 2017); (3) Eddy covariance; and (4) remote sensing methods encompassing landscape and regional scales with satellite image analysis (Talsma, 2018). However, measurements on individual leaves using hand‐held IRGAs/porometers are unable to capture whole plant heterogeneity arising from differences in leaf age, leaf position, plant architecture, as well as temperature and boundary layer (Pearcy et al., 2000). Whole‐plant chambers linked to an IRGA, on the other hand, are costly and replication is time‐consuming (Ryan et al., 2016). In both cases, it is difficult to relate cuvette to field conditions (Tardieu & Simonneau, 1998). Whereas lysimeters are based on soil weight loss and are often unable to accurately separate soil evaporation from *E*. Satellite methods and Eddy covariance can capture water dynamics from field to entire regions, but increasing scale implicitly loses resolution and relies increasingly on assumptions to distinguish *E* from evaporation (Li et al., 2019).

Measuring *E* over time by differences in weight loss using laboratory balances is an integrative, reliable, and easily replicable approach, provided soil evaporation is minimized or accurately accounted for and the measuring time is short enough to avoid detectable gains in dry mass due to CO_2_ assimilation (Cirelli et al., 2012; Cliffton‐Brown & Jones, 1999). Chamber‐based gravimetric measurements of whole plant *E* are rarely reported, probably due to the difficulty in maintaining consistent and stable conditions within the chamber (Fletcher et al., 2007), which can lead to the adjustment of plants to the unstable environmental conditions and subsequently to being misconstrued as “steady‐state” *E* responses.

The difference in water vapor concentration between the leaf intercellular spaces and the atmosphere (represented by the atmospheric water vapor pressure deficit (VPD)) is the driving force for *E*. As a consequence of global warming, VPD has increased (Hatfield & Prueger, 2015). Indeed, values as high as 4.0 kPa associated with heat waves have been reported (Medina et al., 2017). However, assessing the impact of VPD on *E* has been evasive, due to difficulties separating the actual effect of VPD from other climate drivers that vary concomitantly (Grossiord et al., 2020). Assuming water availability in the root zone is not limiting, it follows that high VPD will result in high *E*, due to the increased gradient between the leaf and the atmosphere. Nevertheless, this trend is not always maintained as most species or even genotypes within a species may limit *E* at high VPD via stomatal control of water loss (Bunce, 1984). The apparent stomatal response to VPD is additionally complicated by the possible influence of internal and external factors such as sub‐stomatic CO_2_ concentration, air temperature, light intensity, or hormonal triggers on stomatal behavior (Asch et al., 1995; Bunce, 1997; Yong et al., 1997). The speed with and degree to which a plant responds to VPD has been shown to vary across species and even genotypes (Devi et al., 2010; Ocheltree et al., 2013; Tardieu & Simonneau, 1998). Plant responses of *E* to VPD can roughly be grouped by the hydrostability level against increased evaporative demand (Stocker, 1956). Isohydric, hydrostable plants, such as apple (*Malus pumila*) and rice (*Oryza sativa*), show a pronounced stomatal response to maintain leaf water potential as VPD increases (Jones, 1983), indicated by a nonlinear response to VPD. On the other hand, in anisohydric, hydrolabile plants, such as sunflower (*Helianthus annuus*) and barley (*Hordeum vulgare*), *E* responds mostly linearly to VPD, whereas stomata respond to changes in soil moisture and not to air humidity (Tardieu & Simonneau, 1998). The capacity of a plant to regulate *E* against increased VPD ultimately keeps water in the soil, which may be beneficial if drought conditions occur later in the season (Sinclair et al., 2005). Consequently, determining the independent effect of VPD on *E* for a species or variety will be advantageous when selecting or breeding for drought prone environments.

In this paper, we present a chamber‐based gravimetric method to measure whole‐plant *E* dynamics in a controlled environment in the laboratory, in which VPD can be accurately adjusted, while keeping other environmental drivers controlled. We tested and validated the system using contrasting plant species, differing in life form, habit, and photosynthetic metabolism. The result is an affordable, sensitive, reliable, and highly time‐resolved method of analysis of *E* in response to VPD in combination with environments varying in light intensity and temperature.

## 2 MATERIALS AND METHODS

### 2.1 Description and set‐up of the gravimetric monitoring system for whole‐plant transpiration (MoSysT)

**FIGURE 1:**
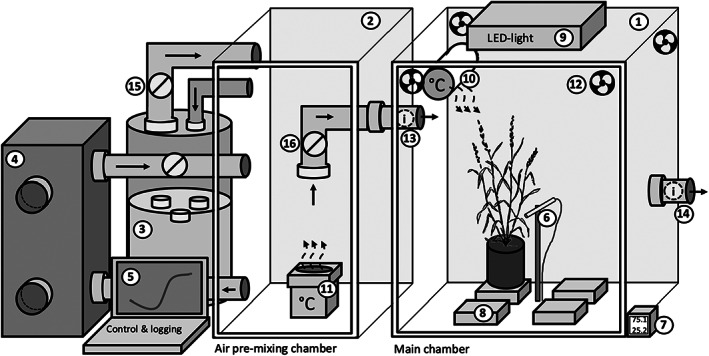
Diagram of the controlled environment gravimetric whole‐plant transpiration monitoring system. The system comprised the following components: (1) Main chamber; (2) Air pre‐mixing chamber; (3) Water tank with floating ultrasonic nebulizer; (4) Dehumidifier; (5) Computer laptop unit/data logging and control software; (6) temperature and humidity sensor; (7) Temperature and humidity logger with visual display; (8) Balances with interface to laptop unit; (9) Light emitting diode; (10) Main chamber heating unit; (11) Air pre‐mixing chamber heating unit; (12) Main chamber air mixing fans; (13) Main chamber inlet control sensors for temperature and humidity; (14) Main chamber outlet control sensors for temperature and humidity, (15) Wet air flow regulator fan, (16) Mixed air flow regulation fan.

MoSysT (Figure 1) consists of two chambers: a main (80 cm wide x 80 cm deep x100 cm high) monitoring chamber (1) and an upstream (66 cm wide x 80 cm deep x100 cm high) air pre‐mixing chamber (2). Floating ultrasonic nebulizers (Fogstar 100, Seliger GmbH, Germany) placed in a 0.1‐m^3^ water tank (3) maintain a wet air depot. A silica‐based rotor dehumidifier (Consorb DC‐10, Seibu Giken, Sweden) provides a constant dry air stream at a flow rate of max. 190 m^3^ h^−1^ (4).

Both air streams discharge into the pre‐mixing chamber before supplying the main measuring chamber from a side entrance. Air streams are forced into the mixing chamber from the wet air depot and into the main measuring chamber by inside‐access‐PVC pipe ventilators (15 and 16) automatically regulated by the control of a custom‐programmed software. Target chamber VPD levels were set by entering the corresponding temperature and humidity values into the software control interface. Based on these target values, the software constantly responds to incoming readings from sensors at 1‐s interval, monitoring humidity and temperature in (13) and out (7) of the main chamber by auto‐adjusting the wet air stream ventilator and heating devices in the main (10) and air pre‐mixing chamber (11) via drive and power regulation, respectively. Air flow into the main chamber was always kept above 40 m^3^ h^−1^ to ensure that the whole chamber volume is exchanged every minute to avoid feedback effects of transpiring plants.

Eight‐band LED lamps (bloom power white 360, Spled GmbH, Germany) installed on top of the main chamber supplied a photosynthetic photon flux density (PPFD) of 1200 μmol m^−2^ s^−1^ 15 cm below the light source (9). Depending on the height of the plants, mean PPFD at the top of the canopy was between 600 and 900 μmol m^−2^ s^−1^. Four individual balances (KERN KB 2400‐2 N d = 0.01 g, with a maximum load of 2400 g), symmetrically arranged to maintain equal light conditions, were placed inside the chamber (8) and connected to a PC (5) via an RS232 port for 1‐minute interval weight recording. Weight losses were considered as transpiration water losses and were standardized to mmol m^−2^ s^−1^ based on the leaf area of the measured plant. To minimize evaporation, the soil surface in the pots was covered with a 2‐cm layer of fine gravel.

To ensure homogenous mixing and turbulent air flux inside the chamber, four adjustable computer box fans were installed near the top of the chamber (12). A combined humidity and temperature sensor (6) placed in the center of the chamber, monitors temperature and relative humidity at the plant level. The sensor was connected to a Tinytag data logger (TV‐4505, Gemini Data Loggers Ltd., UK) logging at 1‐min intervals (14). VPD was calculated using the chamber relative humidity and air temperature values from the Tinytag data loggers as described by Fletcher et al. (2018).

### 2.2 Plant cultivation and post‐chamber measurement and harvest

A group of 10 plant species differing in habit, and photosynthetic metabolism was used (Table 1). All plant species were raised from seeds directly sown into nursery pots filled with standard compost and commercially available fertilized garden soil. After establishment, four seedlings of each species were transplanted into 2‐L plastic pots filled with 700 g soil. Pots were watered every day to maintain well‐watered conditions targeting at 80% of maximum water holding capacity. A complete liquid fertilizer (Wuxal Liquid, AgNova Technologies Pty Ltd, Australia) containing 99 gL^−1^ N; 43 gL^−1^ P; 62 gL^−1^ K; 120 mgL^−1^ B; 10 mgL^−1^ Mo mg L^−1^ Cu; 180 mgL^−1^ Fe; 160 mgL^−1^ Mn; and 60 mgL^−1^ Zn, was applied once a week to ensure full nutrient supply. Plants were grown for 60 days in the greenhouse at the University of Hohenheim, Germany (48°43′N, 09°13′ E, elevation above sea level 407 m). Temperatures ranged between 28 and 32 °C during the day and 20 and 25°C during the night. The air humidity in the greenhouse was between 25% and 30% during the day and between 60% and 65% during night. High‐pressure sodium lamps provided a photosynthetic photon flux (PPF) of 400 to 500 μmol m^−2^ s^−1^, with a 12‐h photoperiod. Before chamber measurements, pots were watered to maximum water holding capacity. For each run, four plants from different species were introduced in the MoSysT and exposed to different VPD environments. Runs were performed once or twice with different plants each run in the same day and for three to four consecutive days. In that way, five to eight plants per species could be measured. After measurements, all leaf blades were cut off the plants and their area determined with leaf area meter (LI‐3000C, LI‐COR, Lincoln, Nebraska, USA), which was used to calculate the transpiration rates on a leaf area basis.

**TABLE 1:** Habit and photosynthetic metabolism, including transpiration rate (*E*) and leaf conductance (*G*_*L*_) of the 10 plant species used in this study

Species	E (mmolm^−2^ s^−1^)	G_L_ (μmolm^−2^ s^−1^)	Photosynthetic metabolism	Habit
*Brachiaria brizantha*	1.40d	79.2d	C4	Stoloniferous grass
*Setaria sphacelata*	1.65d	91.8d	C4	grass
*S. viridis*	2.71 cd	150.0 cd	C4	grass
*Trifolium repens*	2.42d	121.6d	C3	Perennial herbaceous
*Lotus corniculatus*	4.59bc	245.2bc	C3	Perennial herbaceous
*Digitaria eriantha*	2.36d	135.0d	C4	Perennial tufted grass
*Leymus chinensis*	5.88b	307.7b	C3	Perennial bunchgrass
*Phragmatis australis*	4.40bc	233.4	C3	Erect perennial grass
*Chloris virgata*	4.33bc	244.3c	C4	Annual grass
*Kalimeris integrifolia*	9.11a	482.0a	C3	Herbaceous perennial

*Note*: Measurements were carried out at a VPD of 1.7–1.9 kPa. Data were analyzed with a one‐way ANOVA and the Tukey test was used as a post hoc comparison among species. Values followed by the same letters are not statistically significant at *p* < .05.

The transpiration rate was calculated as:

(1) $$E=\Delta_{\text{weight}\left(g\right)}/\left[{\mathrm{MW}}_{H20}*t\left(s\right)*\mathrm{LA}\left(m^{2}\right)*1000\right]$$

where Δ_weight(g)_ is the change in weight between two consecutive measurements; MW_H20_ is the molecular weight of water; t(s) is the time between two consecutive weight recordings in seconds; and LA(m^2^) is the leaf area of the plant in m^2^.

The leaf conductance was calculated according to Buckley (2005):

(2) $$G_{L}=E/\mathrm{VPD}*100$$

where *E* is the transpiration rate as calculated in (Equation 1).

Initially, four species, two common C4 pasture grasses in the tropics and subtropics, *Brachiaria brizantha* (Hochst. ex A. Rich.) Stapf. and *Setaria sphacelata* var. Narok, and two wide‐spread C3 pasture legumes, *Trifolium repens* (L.) and *Lotus corniculatus* L., were used. To test the stability of the system, we exposed the plants to VPDs ranging from 0.5 kPa up to 3.7 kPa. To check whether exposure to the different VPD environments could elicit any acclimation response, we set the VPD values from low to high VPD and then from high to low VPD and *E* was recorded.

Follow‐up runs included six species from three different plant functional groups: two perennial C3 rhizome grasses *Phragmites australis* (Cav.) Trin. ex Steud. and *Leymus chinensis* (Trin). Tzvel; three annual C4 grasses, *Chloris virgata* Swartz, *Setaria viridis* (L.) Beauv. and *Digitaria eriantha* Steud.; and a perennial forb *Kalimeris integrifolia* Turcz. ex DC, a range of VPDs from 0.5 to 2.3 kPa from low to high VPD was used. All other environmental conditions during measurements with the MoSysT were as described earlier.

### 2.3 Statistical analysis and selection criteria of steady‐state *E*

We used a statistical selection criterion to identify the period of constant (steady‐state) whole‐plant transpiration rate. The procedure assumes that leaves pass through a transition period while adapting to any new chamber environment (here consecutively increasing/decreasing levels of VPD) until a steady‐state *E* was reached. We considered a minimum period of 10 min of constant transpiration rate for a given environment as the time requirement to characterize the plants' response.

**FIGURE 2:**
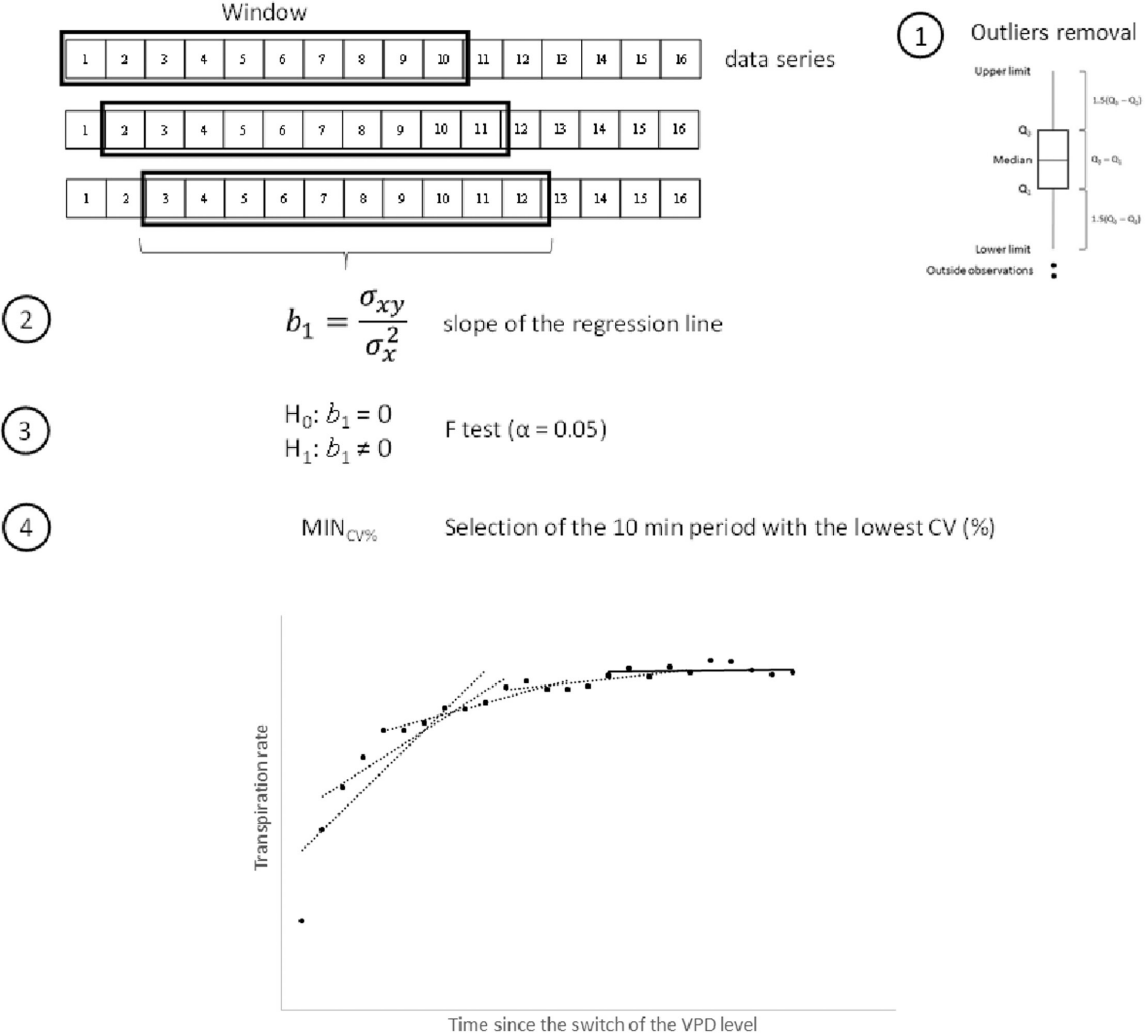
Schematic representation of the algorithm followed to select steady‐state transpiration rates (*E*). (1) Identification and removal of outliers, (2) calculation of the slope of the regression line; (3) Hypothesis system for the selection of slopes not significantly different from zero; (4) Further selection criteria in case more than one 10 consecutive measurements complied with the pre‐requisite in (3).

The steps for identifying and selecting the respective consecutive 10 min periods of steady transpiration rates are summarized in Figure 2. For each chamber environment, which was maintained for at least 30 min, all possible consecutive 10 min intervals were determined. In a first step, each 10 min data subset was analyzed for outliers due to balance disturbances, such as strongly moving leaves in turbulent chamber air fluxes, which would misrepresent the biological response. In case a data point exceeded the upper or lower limits of 1.5‐fold the interquartile range, it was considered as an outlier and discarded from the analysis. For each 10 min period (consisting of 10 single 1‐min plant transpiration rates) of each chamber VPD, a linear regression was fitted. If the slope of the regression line (*b*_1_) was significantly different from zero after an *F*‐test (*p* < .05), the corresponding time interval was considered as a non‐constant plant response period. From those segments which were not significantly different from zero, the interval with the lowest coefficient of variation was selected to represent steady‐state transpiration rate. Having identified and selected a 10‐min period of constant transpiration rates, the 10 values composing the series were averaged and the result was used as the steady‐state *E*. Comparisons of *E* and *G*_*L*_ between species was done in R 3.6.1 with a one‐way ANOVA using the packages *aov* and Tukey's HSD as post hoc comparisons (R Core Team, 2019). To model the response of *E* and *G*_*L*_ to VPD, the SigmaPlot V12.5 regression wizard was used and selection of the best fit was based on the highest *R* ^2^.

## 3 RESULTS

**FIGURE 3:**
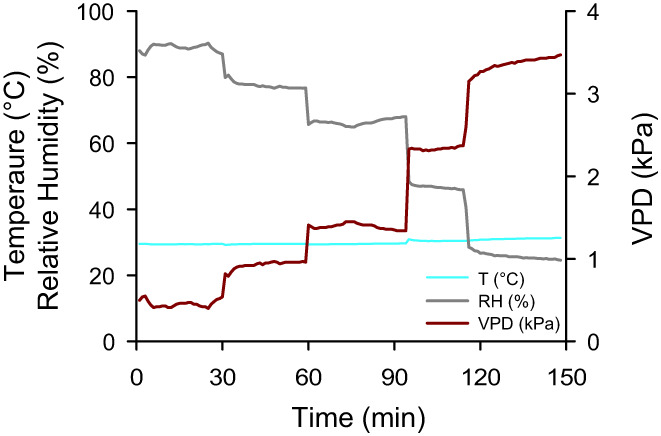
Representative time course of temperature, relative humidity and water vapor pressure deficit (VPD) during transpiration measurements with five successively VPD levels.

MoSysT was able to reach VPD values ranging from 0.5 KPa to 3.7 KPa (Figure 3, Appendix S1), which covers a wide range of conditions including very dry environments. Five different and stable VPD values could be reached and maintained for at least 45 min. The internal adjustment of the system to dry and wet air streams was fast, within 5 min. a new stable VPD was reached in the chamber and could be maintained within +/− 10% of the targeted value (Figure 3). Temperature variation was minimal during measurements and was kept at 29.56 °C +/− 0.58 throughout the whole range of VPDs.

**FIGURE 4:**
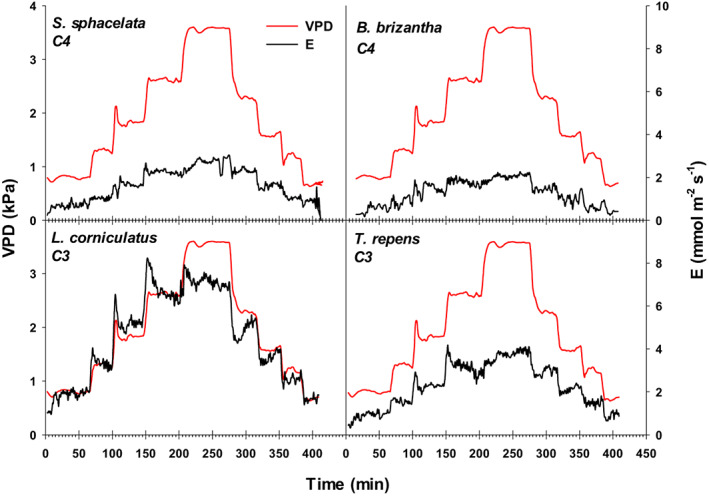
Representative time response of measured VPD and transpiration rates (*E*) transients in the chamber during runs of four plant species: *Brachiaria brizantha, Setaria sphacelata, Trifolium repens*, and *Lotus corniculatus* after changing the VPD settings. Plants were grown under glasshouse conditions for 60 days and transferred to the chamber on the day of measurement.

Depending on species, plants typically reached a steady‐state *E* within 10–20 min after the VPD was changed (Figure 4, Appendix S1). In *T. repens* and *L. corniculatus*, both C3 species, we were able to detect a sudden increase in *E* shortly after each VPD increment in the chamber. This sudden increase lasted between 3 and 5 min after which, a steady‐state *E* corresponding to the specified VPD was reached (Figure 4). This “overshooting” transient response was neither observed in *S. sphacelata* nor in *B. brizantha*.

**FIGURE 5:**
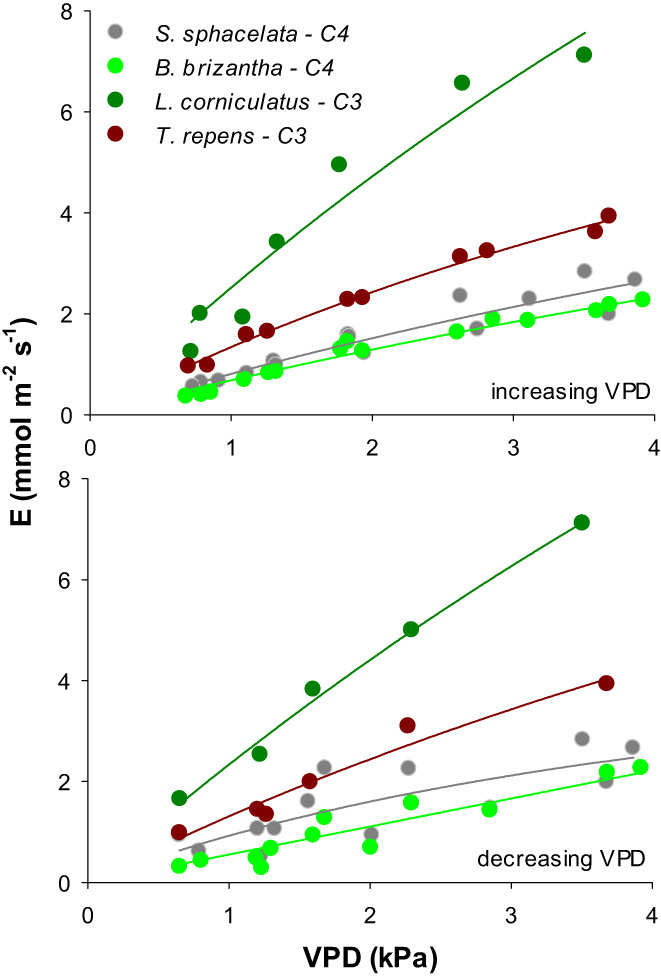
Transpiration rates (*E*) of four species *Brachiaria brizantha, Setaria sphacelata, Trifolium repens*, and *Lotus corniculatus* in response to atmospheric water vapor pressure deficits (VPDs). Plants were grown under glasshouse conditions for 60 days and transferred to the chamber on the day of measurement. The response of *E* to VPD was best fit by a quadratic hyperbolic model in all species (see Appendix S2 for statistical details). VPD values were set from low to high VPD (upper panel) or from high to low VPD (lower panel).

Overall, *E* increased with VPD in all species (Figure 4). At the lowest VPD, *B. brizantha* and *S. sphacelata* transpired around 0.3–0.5 mmol H_2_O m^−2^ s^−1^ and between 2.0 and 2.8 mmol H_2_O m^−2^ s^−1^ at the highest VPD. *T. repens* showed higher transpiration rates (approximately twice as those of *S. sphacelata* and *B. brizantha*) at all VPDs. It started with transpiration rates of 1.0 mmol H_2_O m^−2^ s^−1^ under low VPD and water losses increased to almost 4 mmol H_2_O m^−2^ s^−1^ at a VPD of 3.7. *L. corniculatus* showed the highest transpiration rates among all species, reaching almost 8 mmol H_2_O m^−2^ s^−1^. Transpiration rates at specific VPD values were similar regardless of whether they were obtained by increasing or decreasing VPD (see Appendix S2) and this response was observed in all species. This result can be graphically seen in Figure 5 (Appendices S1 and S2) in which there was no distinction in the response of *E* to VPD regardless of the sequence of VPDs used.

The *E* response to VPD followed a saturation curve and the data were best fit by applying quadratic hyperbolic models. We compared the single hyperbolic rectangular model with the linear model fit, and *r* ^2^ was consistently higher following the nonlinear model (not shown).

**FIGURE 6:**
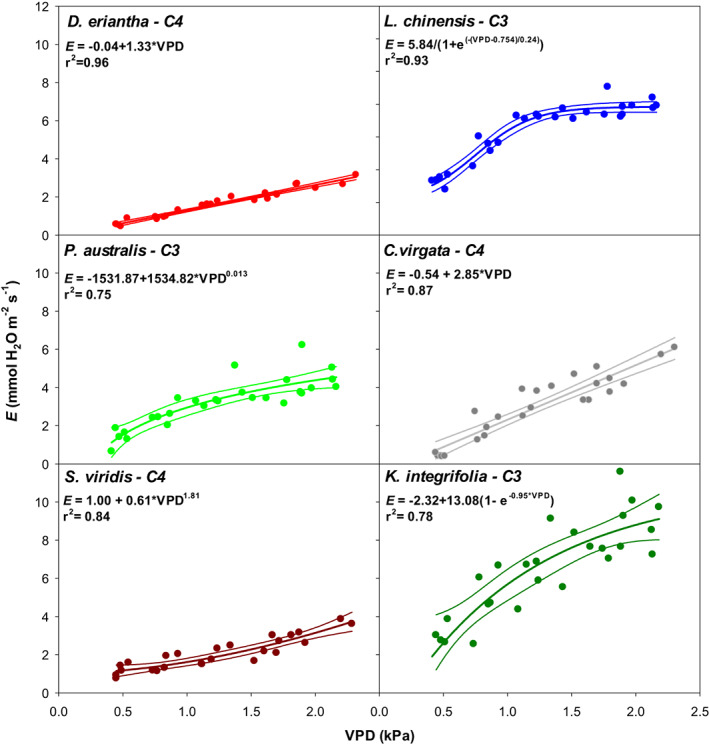
Transpiration rates (*E*) of six species, *Digitaria eriantha, Leymus chinensis, Phragmites australis, Chloris virgata, Setaria viridis*, and *Kalimeris integrifolia*, differing in photosynthetic metabolism, plant habit and functional group in response to atmospheric water vapor pressure deficit (VPD). Plants were grown under glasshouse conditions for 60 days and transferred to the chamber on the day of measurement.

**FIGURE 7:**
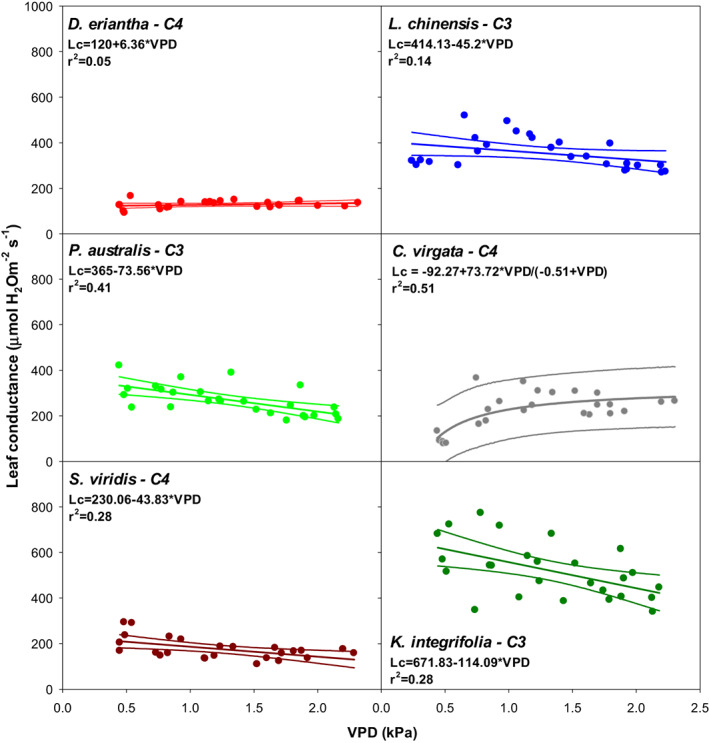
Leaf conductance (*G*_*L*_) of six species, *Digitaria eriantha, Leymus chinensis, Phragmites australis, Chloris virgata, Setaria viridis*, and *Kalimeris integrifolia*, differing in photosynthetic metabolism, plant habit and functional group in response to atmospheric water vapor pressure deficit (VPD). Plants were grown under glasshouse conditions for 60 days and transferred to the chamber on the day of measurement.

As expected C4 species tended to show lower *E* values for each VPD than C3 species (Figure 6, Table 1). The response of *E* to VPD was highly variable and dependent on the species, not only in terms of absolute *E* values attained, but also in the shape of the response to VPD. Whereas in the C4 species the response showed no signs of saturation, in the C3 species on the contrary, a saturating response was observed. Contrarily, leaf conductance was either constant or decreased with VPD (Figure 7, Table 1) in all species but in *C. virgata*, in which leaf conductance increased in a curvilinear fashion in response to VPD.

## 4 DISCUSSION

This study introduced a gravimetric method coupled to an air‐mixing system in which *E* can be accurately determined and environmental conditions were effectively controlled. To our knowledge, this is the first time a fully controlled system is presented that is able to cover a wide range of VPD values from <1 kPa, to almost 4 kPa. It can reproducibly and accurately measure transpiration rate at the whole‐plant level, and thus characterize the response of *E* to short term changes in VPD independently of other environmental drivers such as temperature and light, which have been confounding factors of the impact of VPD on *E*.

The frequency (1‐min interval) of data acquisition allowed the identification of steady‐state rates, as well as the detection in some species, of the sudden initial “overshooting” transpiration response preceding the lower steady‐state transpiration. This response to increases in VPD could be ascribed to the antagonistic effects of simultaneous loss of turgor in guard and subsidiary cells on stomatal aperture and thus *E* (Buckley, 2016; Grossiord et al., 2020). To our knowledge, this phenomenon has been only reported at the single‐leaf level, this is the first time that a similar response is observed at the whole‐plant level. Franks et al. (1997) postulated that any perturbation of the water continuum soil–plant atmosphere, such as rapidly induced soil drying, leaf excision, or drying air, results in a decrease of subsidiary and guard cells turgor pressure, which initially causes stomata to transiently open due to the mechanical advantage of the former, to then close and reach a steady‐state condition. This means that stomata require the amplification of the loss of turgor in the guard cells in order to close in drying air. The nature of the amplifying mechanism is most likely an active one and mediated by ABA (Buckley, 2016). The question remains as to whether the ABA causing stomatal closure is synthesized locally or is the result of a delayed root signaling. The sensibility of MoSysT to these transient responses opens the possibility to further explore the underpinning mechanisms of stomata control on transpiration at the whole‐plant level. Interestingly, the transient increase in *E* was not observed in the grass species used in this study. One might speculate that species‐specific anatomical differences in the stomatal structure (i.e., guard and subsidiary cells) could be responsible as has been pointed out by Franks and Farquhar (2007) and Gray et al. (2020).

One of the main advantages of MoSysT is the control of environmental drivers that can affect *E* concomitantly with VPD; these include not only light and temperature, but also fertilizer supply or soil water content. As typical runs in the MoSysT were shorter than 4 h, possibilities of any significant soil drying eliciting acclimation responses including early stomatal closure could be minimized. This can be noted in the reversibility of the response of *E* to VPD regardless of whether VPD was set from low to high values or in the opposite direction (Figures 4 and 5). Short duration of experiments using the MoSysT also prevent detectable weight gains by photosynthesis in fast‐growing species.

The nonlinearity of the response of *E* to VPD and its reversibility (Figure 4) are two main conditions that support the hypothesis of a direct response of stomata and *E* to VPD regardless of water status of the leaf, the feedforward response of stomatal aperture postulated by Schultze et al. (1972). Nevertheless, the feedforward response can be modulated by the water status of the plant (Schultze & Küppers, 1979) caused, for example, by mid/long‐term soil water deficit. Despite that *E* increases with VPD, restricted or limited *E* at high VPD has been reported in several plant species and genetic variation has been identified in several crop plants (Broughton & Conaty, 2022; Fletcher et al., 2007; Gholipoor et al., 2010; Shekoofa et al., 2014). In most species reported here, particularly C3 ones, limited *E* at high VPD could be also deduced as the best fit for the response to VPD was a saturation quadratic hyperbolic model. Limitation of *E* at high VPD has been postulated as an effective mechanism to increase water use efficiency and to conserve water early in the growing season for use during drought events later in the season and thus it is a desirable trait for crop improvement in drought prone environments.

### 4.1 Concluding remarks

MoSysT is an affordable (total cost is ca. 10,000€) and reliable system that can be used to study the interaction between atmosphere and *E* at the whole‐plant level. It is a simple and scalable system that overcomes several limitations of other gravimetric set ups such as long measuring times and confounding effects due to concomitant variations of other environmental drivers (Devi & Reddy, 2018; Grossiord et al., 2020; Sinclair et al., 2005). Its high resolution in time (recording the weight in 1‐min intervals) allowed the detection of leaf‐level adjustments of *E* to changes in VPD closely.

MoSysT can be easily scaled up either by fitting more balances in a single chamber and/or by replicating the chamber set up, which would allow the use of more complex experimental designs to be tested and thus, increasing our phenotyping capacity.

This has the potential to fill a methodological gap with regard to insight into transpiration dynamics at the mesoscale in response to a key driving factor such as VPD. Further, as light and temperature can be also controlled, studies on impact of different combinations of environmental scenarios can be performed. From the perspective of plant physiology, it can also be combined with long‐ or mid‐term treatments, such as salinity, nutrient deficiency, and drought. Crucial but not well‐understood mechanisms underpinning stomatal aperture dynamics can be closely examined and hypotheses tested. For instance, how diverse is the structure of the guard‐subsidiary cell complex and how determinant it is for the response of *E* to VPD? (Gray et al., 2020). How is the *E* to VPD response modulated by other underlying stressors such as light, high or low temperature, soil water deficit, nutrient deficiencies, salinity? Is the ABA involved in stomatal closure after the “overshooting” response locally synthesized or is it translocated from roots? (Bauer et al., 2013). These and other knowledge gaps can be addressed using the MoSysCT.

## AUTHOR CONTRIBUTIONS

FA and MG devised the concept of MoSysT; MG and MS further developed and constructed and improved the hardware and software to control MoSysT and carried out experiments; AP, MG, and KJ analyzed the data; AP interpreted the results and wrote the manuscript with contributions of KJ, FA, and MG. All authors read and approved the final manuscript.

## FUNDING INFORMATION

This work was funded by Bundesministerium für Wirtschaftliche Zusammenarbeit und Entwicklung; International Grass Network (GrassNet); Deutscher Akademischer Austauschdienst; Baden‐Württemberg Stiftung; Deutsche Gesellschaft für Internationale Zusammenarbeit; Ministerium für Wissenschaft, Forschung und Kunst Baden‐Württemberg; Anton and Petra Ehrmann‐Stiftung.

## CONFLICT OF INTEREST

The authors declare no conflict of interest.

## Supporting information


Appendix S1

Appendix S2

## Data Availability

Data supporting the findings of this study are available in the supplementary data of this article.
