# Supplementary material for: Chamber‐based system for measuring whole‐plant transpiration dynamics
Source: Plant Environ Interact. 2022 Nov 2;3(6):243–53. doi: 10.1002/pei3.10094 (PMC10168032; doi:10.1002/pei3.10094)
Supplement: Supplementary file 2 — Appendix S2 [file PEI3-3-243-s001.docx]

Appendix S2.

Model fitting to Figure 5. The rectangular hyperbolic (E=*a**VPD/(*b*+VPD) model was chosen based on the change in R^2^ as compared to the linear model. To test for differences in *a* and/or *b* depending on the direction of change in VPD, 95% confidence intervals were calculated for each parameter and overlapping of the confidence intervals was used as criterion for significance.

| Species | Model if VPD varies for low to high | Model if VPD varies from high to low | Test for *a* p<0.05 | Test for *b* p<0.05 |
| --- | --- | --- | --- | --- |
| *S. sphatelata* | E=11.347*VPD/(13.10+VPD)  R^2^=0.89 | E=5.96*VPD/(5.44+VPD)  R^2^=0.62 | n.s. | n.s. |
| *B. brizantha* | E=11.69*VPD/(16.07+VPD)  R^2^=0.97 | E=19.78*VPD/(35.68+VP)  R^2^=0.88 | n.s. | * |
| *L. corniculatus* | E=37.42*VPD/(13.84+VPD)  R^2^=0.94 | E=39.18*VPD/(15.76+VPD)  R^2^=0.99 | n.s. | n.s. |
| *T. repens* | E=12.70*VPD/(8.45+VPD)  R^2^=0.99 | E=17.92*VPD/(12.66+VPD)  R^2^=0.96 | n.s. | n.s. |
